# Computerized screening tools for neurocognitive impairment in patients with HIV infection

**DOI:** 10.1186/1471-2334-14-S4-O29

**Published:** 2014-05-29

**Authors:** Ioana-Catrinel Cercel, Șerban Polli, Oana Streinu-Cercel, Anca Streinu-Cercel, Adrian Streinu-Cercel

**Affiliations:** 1 National Institute for Infectious Diseases “Prof. Dr. Matei Balş”, Bucharest, Romania; 2 Carol Davila University of Medicine and Pharmacy, Bucharest, Romania

## 

Neurocognitive impairment is one the non-infectious comorbidities related to HIV infection [1]. An increasing number of studies have shown the importance of assessing attention, motor, memory and cognitive functions under standardized conditions [2]. We have designed a computerized assessment tool, adapting some well-known methods of neurocognitive evaluation, and we have initiated a pilot study to assess the neurocognitive status of HIV-positive patients and controls.

We assessed patients by applying the Motor Screening Task (MST: providing data on the sensorimotor function or comprehension), the Finger Tapping Test (FTT: motor function, self-directed manual motor speed), the Symbol Digit Test (SDT: divided attention, visual scanning, tracking and motor speed), and the Stroop Task (ST: selective attention and cognitive flexibility). All subjects were evaluated with this computerized set of tests during an appointment with the clinical psychologist. For statistical assessment, the T score was used.

We assessed 10 HIV-positive patients and 15 controls. The median age and standard deviation were 26.5±11.5 years (range: 23-54) in the HIV group and 34±8 years (range: 25-53) in the control group. The MST scores were 720±131 ms and 629±79 ms in HIV vs. controls. The FTT scores were 167±24 ms and 163±12 ms in HIV vs. controls. The SDT scores were 6646±2905 ms and 2994±681 ms in HIV vs. controls. The ST scores were 689±92 ms, 1602±1009 ms and 2045±580 ms in the HIV group and 486±118 ms, 1041±244 ms and 1023±163 ms in the control group. Despite the low number of subjects included in the study, the T score showed a statistically significant difference between the two groups for MST, SDT, ST; no statistically significant difference was observed for FTT between the two groups.

This pilot study has shown that in HIV-positive patients, the cognitive deficit tends to appear earlier than the motor one. Larger studies are needed to confirm these preliminary results and to provide more information on the clinical impact of this computerized tool for neurocognitive screening.
